# Overview of Potential Clinical Applications of Hemoglobin Vesicles (HbV) as Artificial Red Cells, Evidenced by Preclinical Studies of the Academic Research Consortium

**DOI:** 10.3390/jfb8010010

**Published:** 2017-03-15

**Authors:** Hiromi Sakai

**Affiliations:** Department of Chemistry, Nara Medical University, 840 Shijo-cho, Kashihara, Nara 634-8521, Japan; hirosakai@naramed-u.ac.jp; Tel.: +81-744-29-8810

**Keywords:** blood substitutes, artificial red cells, oxygen carriers, hemoglobin, liposomes, hemoglobin (Hb)-based oxygen carriers (HBOCs), resuscitative fluid, organ perfusate, carbon monoxide, photosensitizer

## Abstract

Hemoglobin (Hb) is the most abundant protein in whole blood. This fact implies that the oxygen binding and releasing function of Hb is the most vital for sustaining life. All Hb is compartmentalized in red blood cells (RBCs) with corpuscular Hb concentration of about 35 g/dL, covered with a thin biomembrane. In spite of its abundance, Hb sometimes shows toxicity once it is leaked from RBCs. The shielding effect of the RBC membrane is physiologically important. Based on this structural importance, we have studied artificial red cells (Hb vesicles, HbV) as artificial oxygen carriers, which encapsulate a purified and concentrated Hb solution in phospholipid vesicles, mimicking the cellular structure of RBCs. Our academic research consortium has clarified the safety and efficacy of this HbV, aiming at clinical applications. Because of some superior characteristics to those of RBCs, HbV has the potential for use not only as a transfusion alternative but also for oxygen and carbon monoxide therapeutics, perfusate for transplant organs, and photosensitizer. In this review paper, such potential applications are summarized.

## 1. Introduction

**Figure 1:**
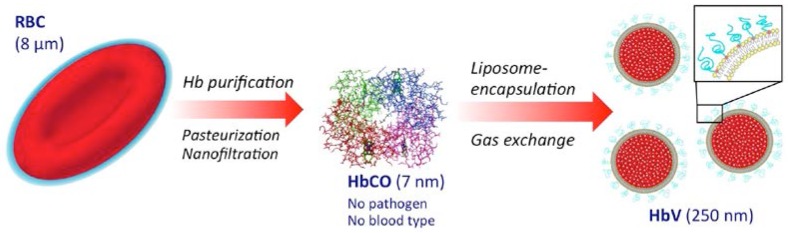
Preparation of hemoglobin vesicles (HbV) from outdated NAT (nucleic-acid amplification testing)-inspected red blood cells (RBC) provided by the Japanese Red Cross. The HbCO purification procedure includes pasteurization and nanofiltration for utmost safety from infection. Liposome encapsulation shields the toxic effects of molecular hemoglobin (Hbs).

The established blood donation and transfusion system has contributed to human health and welfare. Nevertheless, the system could be greatly improved; (i) if the pathogen contamination could be eliminated completely; (ii) if the blood type antigen on the red blood cell (RBCs) surface could be removed completely and the resulting RBCs could be supplied as universal blood; (iii) if the donated blood could be stored for years at ambient temperature; and (iv) if the donated blood could be useful whenever and wherever it is required without a cross-matching test and without fear of infection; (v) It is also important to maintain a sufficient number of donors to support the system. To address such challenges and to support the present blood donation and transfusion system, hemoglobin (Hb)-based oxygen carriers (HBOCs) of various kinds have been developed as a transfusion alternative [1]. Several HBOCs such as intramolecular cross-linked, polymerized, and polymer conjugated Hbs have been tested in clinical phase studies but the cell-free structures of these chemically modified HBOCs retained some side effects of molecular Hbs, such as renal toxicity, vasoconstriction, hypertension, higher incidence of infarction, and death [2]. These results imply the importance of mimicking the cellular structure of RBCs to shield the toxic effects of molecular Hbs. Ultrathin membranes of polymer and cross linked protein membrane artificial red blood cells containing Hb and enzymes were prepared in 1964 [3]. Studies of encapsulation of functional molecules with phospholipids started after the discovery of liposomes by Bangham in the 1960s [4]. Djordjevici and Miller in 1977 first reported liposome encapsulated Hb (LEH) [5]. Many research groups have attempted encapsulation of Hb using liposomes, improving the biocompatibility, stability during storage, and oxygen-carrying capacity (Figure 1). Because of the difficulty in resolving the issues above and because of the need for large-scale production, most groups terminated the development. However, our academic consortium has continued the research and development of hemoglobin vesicles (HbV) since late Emeritus Prof. Tsuchida started in the 1980s. Considerable efforts have been undertaken to attain the present formulation of HbV [6].

## 2. Preparation, Characteristics and Biocompatibility of HbV

In Japan, the research and development of HBOCs began in the 1980s with the concept of recycling of unused donated blood. The former concept of using Hb from outdated RBCs was based on the preservation of other glycolytic and metHb reducing enzymes present in RBCs. However, our present concept is to eliminate such unstable enzymes during virus inactivation and removal processes for the utmost safety from infection, even though the donated blood was confirmed as virus-free by specific nucleic acid amplification tests. The processes of Hb purification from outdated donated human blood includes procedures of pasteurization (60 °C, 12 h) and nanofiltration, respectively, for virus inactivation and removal. The concentrated and purified carbonyl hemoglobin (HbCO) solution (35–40 g/dL) is encapsulated with liposomes comprising four lipids: 1,2-dipalmitoyl-*sn*-glycero-3-phosphatidylcholine (DPPC), cholesterol, 1,5-*O*-dihexadecyl-d-glutamate, and 1,2-distearoyl-*sn*-glycero-3-phosphatidylethanolamine-*N*-PEG_5000_. These lipids are selected in terms of stability, encapsulation efficiency, and biocompatibility [6]. The oxygen affinity (P_50_) of HbV is adjusted by co-encapsulation of an allosteric effector, pyridoxal 5′-phosphate, to 9–30 Torr, depending on the usage. The particle size is adjusted to 250–280 nm by extrusion or kneading. Small-angle X-ray scattering (SAXS) clarified the spherical unilamellar structure (one bilayer membrane) encapsulating a concentrated Hb solution [7]. HbCO can be converted to HbO_2_ by illuminating visible light under an aerobic atmosphere. Finally, the deoxygenated HbV is purged with nitrogen in vials for long-term storage. In the case of using HbV as a CO carrier, carbonyl state HbV without the processes of decarbonylation and deoxygenation is purged with CO gas. The Hb concentration is adjusted to 10 g/dL, which is slightly lower than that of human blood (12–15 g/dL), but much higher than the transfusion trigger, known as 6–7 g/dL in critical patient blood. Usually, HbV is suspended in a physiological saline solution (0.9% NaCl), but it can be suspended in a phosphate buffered saline or any colloidal solution such as 5% albumin solution for clinical use. The percentage of the occupied volume of the HbV particles corresponds to about 40%–45% (c.f., hematocrit of blood is about 40%–55%). Therefore, the suspension is a concentrated particle dispersion, similar to RBCs in blood.

Encapsulation can shield the toxic effects of molecular Hbs [8,9,10]. However, the biocompatibility of the materials for encapsulation, e.g., the lipid membrane, must be considered. The present lipid formulation of HbV shows no significant effects on the complement system, immunological response, blood coagulation, platelet function, kallikrein–kinin, hematopoiesis, etc. [11,12,13,14,15,16]. The HbV particles are finally captured by the reticuloendothelium system (RES). Therefore, transient splenohepatomegaly is observed depending on the dosage of HbV [17,18]. During storage, HbV is stable for over two years at room temperature. During blood circulation, HbV does not rupture. The particles maintain their integrity [19]. After HbV is degraded in RES, the decomposed materials are excreted in urine and feces.

## 3. Potential Usage of HbV as a Transfusion Alternative

**Figure 2:**
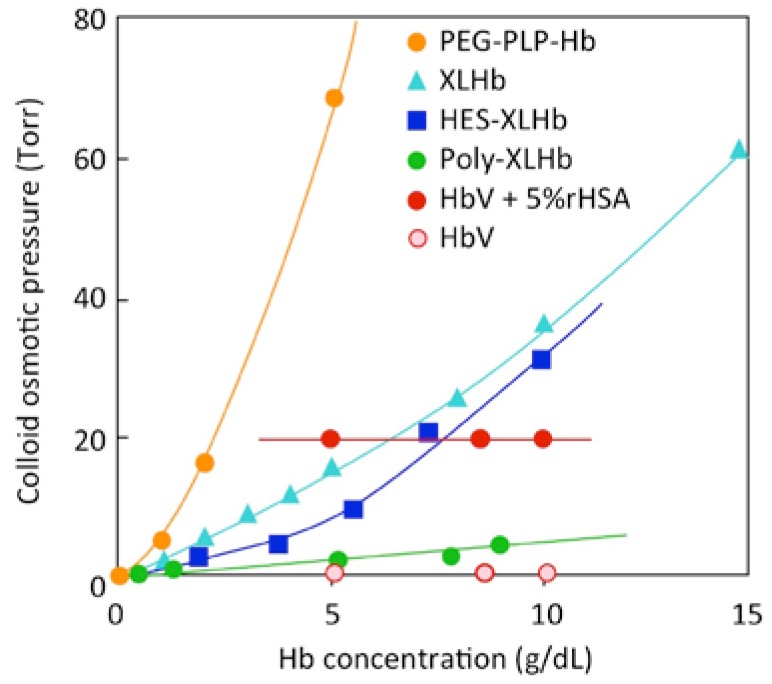
Colloid osmotic pressure (COP) of solutions of various Hb-based oxygen carriers. Chemically modified Hb solutions show concentration-dependent COP because of the colligative property of homogeneous macromolecular solution, and COP exceeds physiological values (20–25 Torr) at higher Hb concentrations. However, HbV as well as RBC shows no COP. When HbV is suspended in human serum albumin (HSA), it shows physiological COP values, cited partly from [19], independent of the Hb concentration.

One important characteristic of HbV is that the suspension of HbV shows no colloid osmotic pressure [20] (Figure 2), similarly to RBC when suspended in saline solution. Plasma proteins in blood, mainly albumin, contribute to the colloid osmotic pressure, about 20–25 Torr, which is important to maintain equilibrium of water contents between blood and interstitial tissue. In the case of massive blood loss, restoration not only of the oxygen-carrying capacity, but also of blood volume is important. Blood volume is first restored by injection of the crystalloid solution, followed by injection of a plasma substitutes such as albumin, hydroxyethyl starch, modified fluid gelatin, or dextran. When the blood Hb level declines below a transfusion trigger, 6–7 g/dL, an RBC concentrate is transfused to restore the oxygen-carrying capacity. This is the basic protocol of RBC transfusion at massive hemorrhage, minimizing the usage of allogeneic transfusion. In the case of HbV, it is possible to follow the protocol and to inject HbV in place of RBC [21], or inject HbV from the beginning of resuscitation. HbV can be mixed with a plasma expander before injection to provide a physiologically appropriate colloid osmotic pressure to the HbV suspension. This is a quite important point because a chemically modified Hb solution shows colloid osmotic pressure depending on its own concentration. When the Hb concentration of a chemically modified Hb solution is approximately 10 g/dL, the colloid osmotic pressure greatly exceeds the physiological pressure. However, both RBC and HbV suspended in saline possess no colloid osmotic pressure.

Table 1 presents results of animal experiments of HbV to confirm the potential usage of HbV as a transfusion alternative. In most cases, HbV were suspended in 5% HSA or recombinant rHSA in advance to adjust colloid osmotic pressure. Results confirmed that over 90% exchange transfusion with HbV suspended in HSA sustained the hemodynamics and blood gas parameters, indicating that HbV surely carries oxygen even at a low hematocrit [22]. Microhemodynamics and tissue oxygenation were observed after hemodilution with HbV [23,24]. Isovolemic hemodilution in rats was achieved by repeating 1 mL blood withdrawal from an artery and 1 mL HbV intravenous injection [15,22]. This procedure of hemodilution might correspond to repeated injection of HbV at repeated hemorrhage without induction of a shock state.

In the case of hemorrhagic shocked animals, HbV suspended in HSA showed a sufficient resuscitative effect [25,26,27,28,29,30,31]. Repeated resuscitation at a repeated shock state of massive hemorrhage was tested with rabbits [26,27]. The beagle dogs survived for over 1 year and the rats for over 14 days after resuscitation from hemorrhagic shock without major side effects except the transient splenomegaly, with an increase in plasma cholesterol levels attributable to the RES trap of HbV and succeeding degradation [28,31]. Recovery of hematocrit was confirmed. It is noteworthy that one-year-stored HbV showed effective resuscitation without showing acute lung injury [29]. Continuous injection of HbV into rats with hemorrhagic shock and uncontrolled hemorrhage showed survival even after hematocrit decreased to less than 2% [32].

Cardiac surgery using extracorporeal membrane oxygenator (ECMO) requires a fluid to prime the circuit, resulting in the dilution of blood. The effect of hemodilution to the neurological function of the newborn patient is significant because the decreased oxygen supply during surgery affects the brain function. Damage appears after the newborns are grown up. It was confirmed using a rat model that ECMO primed with HbV suspended in HSA showed sustained oxygenation and prevented neurocognitive decline [33].

## 4. Potential Usage of HbV as an Oxygen Carrier for Ischemic Disease and Ex Vivo Perfusion, a Photosensitizer, etc.

**Figure 3:**
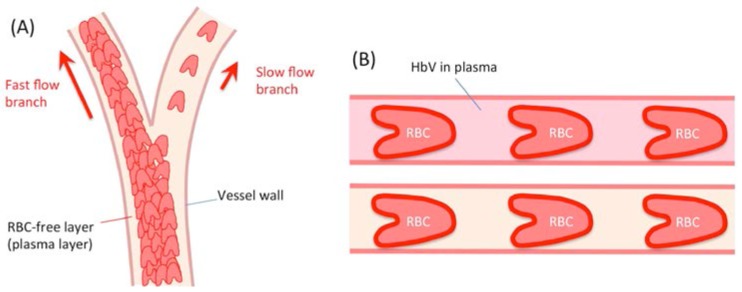
Schematic representation of blood flow in an arteriole and a capillary. (**A**) An arteriole with a bifurcation shows different blood distributions to daughter branches when the branches show different blood flow rates, causing plasma skimming. HbV distributes homogeneously in plasma phase and contributes to increased Hb content in the daughter branch of lower blood flow; (**B**) RBCs (8 µm) flow through a capillary (about 5 µm diameter). Injected HbV are expected to be distributed homogeneously in the plasma phase and to increase the total contents of Hb in the capillary.

Microscopic view of peripheral blood flow demonstrates the heterogeneous distribution of RBCs in blood vessels (Figure 3). Because of the centralization of RBCs in blood vessels, especially in fast blood flow in arterioles, an RBC-free layer (plasma layer) is formed near the vessel wall. At a bifurcation of arterioles where blood flow rates of two daughter branches are extremely different, plasma skimming is induced, resulting in different distribution of RBCs to the two branches. The lowered RBC distribution is expected to be significant at the entrance to the pathological tissues of circulation disorder such as infarction. In normal capillaries, hematocrit is lower than the systemic hematocrit because of the Fahraeus effect. As a result, the distance between the aligned RBCs in the narrow capillaries lengthens. In such situations, injection of HbV is expected to improve tissue oxygenation because of the following reasons.

One important benefit of HbV is the smaller particle size (230–280 nm) than RBC (8 µm). HbV is dispersed homogeneously in the plasma phase after administration into blood circulation. As a result, the HbV concentration after plasma skimming and in normal capillaries becomes higher in theory than the systemic HbV concentration. In a condition where plasma flow is retained in spite of suppressed RBC supply, the presence of HbV in plasma can sustain the oxygen supply to peripheral tissues. This concept supports the efficacy of HbV as oxygen therapeutics for oxygenation of ischemic brain tissues [34], skin flap ischemic tissues [35,36], pre-eclampsia [37], etc. (Table 2). Oxygen affinity of HbV can be adjusted by co-encapsulation of an allosteric effector such as pyridoxal-5′ phosphate (PLP). Without PLP, the oxygen affinity (P_50_) of HbV becomes 9 Torr. This left-shifted HbV is advantageous to target oxygen to ischemic tissues, whereas, in normal tissues, HbV does not release oxygen. This is a concept of targeted oxygen delivery [35,36]. A high O_2_-binding affinity (lower P_50_) might also be effective for improving the O_2_ saturation of Hb in pulmonary capillaries when exposed to a hypoxic atmosphere or with an impaired lung function. The viscosity of HbV suspended in HSA is about 3 cP. Some plasma substitutes cause flocculation of HbV and hyperviscosity. Hyperviscosity, however, would not necessarily be deleterious in the body and might even be beneficial for peripheral perfusion in some cases such as skin flap ischemia for improved functional capillary density and higher shear stress on the vascular wall that would induce vasorelaxation.

Systemic administration of HbV can elevate tumor tissue oxygen tension. The combination of irradiation therapy reduces the tumor size [38]. Tumor capillaries are irregularly shaped and narrow in comparison to normal capillaries. Therefore, hematocrit is much lower than normal. Small-sized HbV dispersed in the plasma phase can permeate through such irregular capillaries. Because of the junction gap separating the endothelial cells, the vascular wall is leaky. Small particles such as HbV permeate to the tumor cells in a phenomenon called the enhanced permeability and retention (EPR) effect.

Positron emission computerized tomography using ^15^O_2_ is an effective tool to measure the cerebral metabolic rate of oxygen (CMRO_2_) for diagnosis of brain infarction. Inhalation of ^15^O_2_ gas presents substantial problems such as difficulties in handling radioactive gas, exposing the patient and hospital staff to radioactivity. Injectable systems of ^15^O_2_-carrier are expected to solve such problems. Injection of ^15^O_2_-HbV has been proven to be effective for CMRO_2_ to detect rat brain infarction [39,40].

Organ preservation should be improved by supporting oxygen metabolism by perfusion with a fluid such as oxygen-carrying blood. It has been confirmed that a dissected and immersed mouse intestine perfused arterially with HbV suspended in HSA at 37 °C maintained peristaltic motility and tissue intactness for over 2 h, and enabled ex vivo observation of intestinal function. This was the first attempt to test HbV as an organ perfusate [41]. Araki et al. tested perfusion of amputated rat hind legs with HbV suspended with ET-Kyoto fluid for 6 h and successive replantation of the leg. The rat used the replanted leg to walk at 3 months [42]. These results strongly support the potential for use of HbV as an oxygen-carrying organ preservation fluid for other vital organs, and for oxygen-carrying cell culturing media to maintain aerobic metabolism of the cells [43].

Rikihisa et al. reported utilization of HbV as a photosensitizer, a target of laser treatment of port-wine stain (capillary malformation) [44], because injection of HbV can increase capillary Hb levels effectively as depicted in Figure 3B, producing more heat and photocoagulation.

The situation ‘cannot ventilate, cannot intubate’ during the induction of anesthesia is one of the most serious complications. It was recently clarified that HbV injection can prolong the time to circulatory collapse during apnea [45].

## 5. Potential Usage of HbV as a Carbon Monoxide Carrier

Motterlini et al. synthesized a series of CO-releasing metal complexes and discovered some therapeutic benefits such as a cytoprotective effect in animal models of hemorrhagic shock, septic shock, and ischemia reperfusion injury [46]. CO-bound RBC has been tested for ischemia reperfusion injury at hemorrhagic shocked animals [47]. CO-HbV was first tested for resuscitation from hemorrhagic shock in a rat model [48] (Table 3). Shock was induced by withdrawing 50% of circulating blood volume. Isovolemic CO-HbV suspended in HSA was injected after 15 min. It is particularly interesting that the blood HbCO level decreased in 3 h, and that the dissociated CO appeared in the exhaled air. Aspartate aminotransferase (AST) and alanine aminotransferase (ALT) levels were reduced as compared to O_2_-HbV injection, indicating that CO showed cytoprotective effects against reperfusion injury. It is well-known that CO binds to Hb 200 times more strongly than O_2_ does. In blood circulation, however, O_2_ is more abundant than CO, and CO tends to be dissociated from Hb and exhaled through the lung with time. No toxicological symptom was observed during the experiment in spite of the large dosage. Otagiri and Maruyama et al. applied CO-HbV for models of bleomycin-induced pulmonary fibrosis [49], dextran sulfate sodium-induced colitis [50], and acute pancreatitis [51]. Liberated CO showed significant anti-inflammatory and anti-oxidative properties, probably attributable to the interaction of CO with hemeproteins related to the production of reactive oxygen and nitrogen species in the body in pathological conditions.

From the viewpoint of production, CO-bound HbV is more easily produced for storage than deoxygenated HbV because the processes of decarbonylation and deoxygenation can be omitted. After releasing CO in blood circulation, HbV reversibly binds O_2_, and becomes an oxygen carrier. CO-HbV is expected to provide a unique opportunity for the clinical treatment of various pathological conditions.

## 6. Conclusions

In addition to the efficacy evaluations summarized in this paper, we have reported numerous safety evaluations ([52], and references therein), all of which assure the biocompatibility of HbV even at a high dosage. The research of HbV was initiated aiming at a transfusion alternative. However, HbV now has the potential to be used for clinical situations other than transfusion. The estimated marketability in the future is expected to be greater than that anticipated earlier. With strong funding support from Japan Agency for Medical Research and Development, we are struggling to start clinical studies aimed at the eventual realization of HbV for clinical usage.

## Figures and Tables

**Table 1:** Usage of HbV as a transfusion alternative (substitute for RBC transfusion).

Application	Animal Species	Brief Description of Main Results	References
Isovolemic hemodilution (repeated injection at hemorrhage)	Wistar rats	90% blood exchange with HbV suspended in HSA showed stable hemodynamics	[22]
	Syrian golden hamsters	80% blood exchange with HbV suspended in HSA showed stable hemodynamics and microvascular responses	[23,24]
	Wistar rats	40% blood exchange with HbV suspended in rHSA, and 14 days observation	[15]
	Wistar rats	60% blood exchange with a plasma expander (high Mw HES, low Mw HES, MFG, or rHSA) and subsequent injection of HbV (20 mL/kg)	[21]
Hemorrhagic shock	Wistar rats	50% blood withdrawal and resuscitation, 6 h observation	[25]
	Japanese white rabbits	Twice of 40% blood withdrawal and resuscitation	[26]
	New Zealand white rabbits	Withdrawing blood to a mean arterial blood pressure of 30–35 mm Hg, and resuscitation with HbV/rHSA	[27]
	Wistar rats	50% blood withdrawal and resuscitation, 14 days observation	[28]
	Lewis rats	40% blood withdrawal and resuscitation, 6 h observation showed absence of acute lung injury. One-year-stored HbV was used for resuscitation	[29]
	Beagle dogs	50% blood withdrawal and resuscitation. 4 h observation of hemodynamics	[30]
	Beagle dogs	40% blood withdrawal and resuscitation, one year safety observation	[31]
Uncontrolled hemorrhage	Wistar rats	Animals were heparinized and bled continuously from caudal artery. Injection of HbV suspended in HSA extended survival	[32]
Priming of ECMO	Wistar rats	Use of HbV for cardiopulmonary bypass priming prevented neurocognitive decline	[33]

**Table 2:** Usage of HbV as an oxygen carrier for oxygen therapeutics and diagnosis, and as a photosensitizer and an ex vivo perfusate.

Application	Animal Species	Brief Description of Main Results	References
Brain ischemia	Wistar rats	HbV injection to a middle cerebral artery occlusion/reperfusion model reduced cerebral infarct volume. HbV injection to an arachidonic acid-induced stroke model improved motor dysfunction score and suppressed edema	[34]
Skin flap ischemia	Syrian golden hamsters	Dorsal skin flap oxygenation was improved by systemic application of a highly viscous left-shifted HbV (P_50_ = 9 Torr)	[35]
	DDY mice	Dorsal skin flap oxygenation and wound healing was improved by systemic application of left-shifted HbV (P_50_ = 9 Torr)	[36]
Pre-eclampsia	Wistar rats	L-NAME was infused intravenously for 7 consecutive days between gestational day 14 (G14) and G21 to prepare a pre-eclampsia model with narrow placental spinal artery remodeling and impaired fetal growth. Co-injection of HbV improved fetal oxygenation and growth	[37]
Tumor	C57BL/6 mice	Lewis lung carcinoma in the left hind leg of mice. HbV administration increased tumor tissue oxygen tension and, following 20-Gy irradiation, delayed tumor growth	[38]
^15^O-PET	Sprague Dawley rats	^15^O_2_-HbV was injected to measure the cerebral metabolic rate of oxygen for diagnosis of brain infarction	[39,40]
Organ perfusion	BALB/c mice	Ex vivo arterial perfusion of intestine with HbV/HSA for 2 h maintained peristaltic motion	[41]
	Wistar rats	Amputated hind limb was perfused with HbV/ET-kyoto for 6 h and re-planted. The rat used the replanted leg after 3 months	[42]
Cell culturing	Rat hepatocyte	Culturing rat hepatocytes with HbV in 2D flat-plate perfusion bioreactor	[43]
Photo-sensitizer	Chicken wattle as a model of port-wine stain	Injection of HbV increases the capillary content of total Hb as a target of dye laser treatment. It would produce more heat and photocoagulation by the treatment	[44]
Apnea	Sprague Dawley rats	Injection of HbV prolonged the time to circulatory collapse during apnea in anesthesia	[45]

**Table 3:** Usage of HbV as a CO carrier for anti-inflammatory and anti-oxidative effect.

Application	Animal Species	Brief Description of Main Results	Reference
Hemorrhagic shock	Wistar rats	Hemorrhagic shocked rats were resuscitated with CO-HbV suspended in HSA. AST and ALT levels were reduced as compared to O_2_-HbV injection	[48]
Pulmonary fibrosis	Sea-ICR mice	Bleomycin-induced pulmonary fibrosis mice that received CO-HbV showed suppression of progression of fibrosis and improved respiratory function	[49]
Colitis	Sea-ICR mice	Dextran sulfate sodium-induced colitis model mice receiving CO-HbV improved colitis symptoms, colonic histopathological changes and the duration of survival compared to both saline and O_2_-HbV administration	[50]
Pancreatitis	BALB/cN mice	Pancreatitis model mice were prepared with a choline-deficient ethionine-supplemented diet. CO-HbV inhibited the production of systemic proinflammatory cytokines, neutrophil infiltration, and oxidative injuries	[51]
